# Bi-Directional Evidence Linking Sentence Production and Comprehension: A Cross-Modality Structural Priming Study

**DOI:** 10.3389/fpsyg.2019.01095

**Published:** 2019-05-28

**Authors:** Kaitlyn A. Litcofsky, Janet G. van Hell

**Affiliations:** ^1^Aphasia and Neurolinguistics Research Laboratory, Department of Communication Sciences and Disorders, Northwestern University, Evanston, IL, United States; ^2^Bilingualism and Language Development Lab, Department of Psychology, Pennsylvania State University, University Park, PA, United States

**Keywords:** cross-modality structural priming, production, comprehension, event-related potentials, sentence processing

## Abstract

Natural language involves both speaking and listening. Recent models claim that production and comprehension share aspects of processing and are linked within individuals (Pickering and Garrod, 2004, 2013; MacDonald, 2013; Dell and Chang, 2014). Evidence for this claim has come from studies of cross-modality structural priming, mainly examining processing in the direction of comprehension to production. The current study replicated these comprehension to production findings and developed a novel cross-modal structural priming paradigm from production to comprehension using a temporally sensitive online measure of comprehension, Event-Related Potentials. For *Comprehension-to-Production* priming, participants first listened to active or passive sentences and then described target pictures using either structure. In *Production-to-Comprehension* priming, participants first described a picture using either structure and then listened to target passive sentences while EEG was recorded. *Comprehension-to-Production* priming showed the expected passive sentence priming for syntactic choice, but not response time (RT) or average syllable duration. In *Production-to-Comprehension* priming, primed, versus unprimed, passive sentences elicited a reduced N400. These effects support the notion that production and comprehension share aspects of processing and are linked within the individual. Moreover, this paradigm can be used for the exploration priming at different linguistic levels as well as the influence of extra-linguistic factors on natural language use.

## Introduction

In natural language use, individuals speak in order to communicate their ideas and listen in order to gather new information. However, though individuals are engaged in both production and comprehension processes in daily dialogue, most psycholinguistic research on production has remained largely insular from research on comprehension, and vice versa, as exemplified by classical psycholinguistic models focusing exclusively on word production (e.g., Levelt, 1999) or word recognition (e.g., McClelland and Rumelhart, 1981). Recent theories (Pickering and Garrod, 2004, 2013; MacDonald, 2013; Dell and Chang, 2014), though, contend that production and comprehension actually share their underlying representations or processing mechanisms. In line with these theories, structural priming, specifically cross-modality structural priming (e.g., Bock, 1986; Segaert et al., 2012) empirically demonstrates that the two modalities share aspects of processing. Without shared representations or underlying processing mechanisms, no priming from one modality to the other would be present. These cross-modality structural priming studies typically examine the influence of comprehension on target production processing. The current study replicated this comprehension-to-production priming, but extended the paradigm to study whether the link between the two modalities is also evident when examining the influence of production on subsequent comprehension processing, in a novel cross-modality structural priming methodology using behavioral and event-related potential techniques (ERPs). Thus, the main goal of this study was to determine whether there is a measurable impact of processing in one modality on the other, using the tool of structural priming. Specifically, we focused on the interaction of comprehension and production in the less-studied direction, from production-to-comprehension (and compare that to priming in the reverse direction), using ERPs to obtain temporally sensitive measures of online sentence comprehension.

First, we will discuss the dissociation between production and comprehension, and recent models aiming to link these two processes, then we will turn to structural priming focusing on cross-modality structural priming and how that technique has been applied to study language production and comprehension and their interaction, and then finally, introduce the current study.

### Production and Comprehension

The separate evolution of production and comprehension research has several roots. First, psycholinguistics has been influenced by the idea that language enjoys a privileged and a modular instantiation in the mind and brain (Chomsky, 1965; Fodor, 1983), though more recent neuroimaging work suggests that language and cognitive processing is subserved by highly interconnected networks (e.g., Bressler and Menon, 2010) and that at least some aspects of language comprehension and language production are supported by domain-general networks (e.g., Ullman, 2001; Abutalebi and Green, 2008; Hagoort, 2013). Second, production and comprehension have been considered to be separate processes as children often can understand syntactic structures before they can produce them (e.g., Fraser et al., 1963), though others argue that the two modalities rely on similar processing mechanisms that are applied to different contexts (e.g., Kempen et al., 2012; Hagoort and Meyer, 2013). Finally, experimental research tends to focus only on production or on comprehension, due to research paradigms designed around the classical theories that focus exclusively on production (e.g., Levelt, 1999) or on comprehension (McClelland and Rumelhart, 1981), and to methodological ease (i.e., labs that are optimized for research on comprehension may not be as well-equipped to conduct production research, and vice versa).

Recently, several theories have been proposed that link the processes of production and comprehension (Pickering and Garrod, 2004, 2013; MacDonald, 2013; Dell and Chang, 2014). MacDonald’s (2013) Production-Distribution-Comprehension theory (PDC) links production and comprehension at the community level by arguing that the nature of utterances are shaped by speakers minimizing their production demands, and comprehenders’ processing becoming attuned to the specific distribution of utterances in the community. However, this theory does not specifically link production and comprehension processing within an individual. In contrast, the P-Chain model (Dell and Chang, 2014) and Pickering and Garrod’s (2013) integrated account of production and comprehension more explicitly link production and comprehension processes within an individual, and emphasize the role of prediction. Dell and Chang’s (2014) P-Chain model states that during language comprehension, listeners make predictions of the upcoming input. Since this prediction is top-down, Dell and Chang label it as a production process. Therefore, prediction processes link production and comprehension during comprehension processing only and would predict the observation of a relationship between modalities only in the direction of comprehension to production. Relatedly, Pickering and Garrod (2013) claim that forward prediction models are created and used to facilitate processing during both production and comprehension, thus linking both modalities during both comprehension and production processing. However, the theory suggests that production and comprehension make use of separate representations, and that the prediction models are separate as well. Therefore, cross-modality priming may not by expected, unless it is possible that the prediction models influence comprehension and production processing. Finally, Pickering and Garrod’s (2004) Interactive Alignment model focuses on how production and comprehension processes are related across individuals in dialogue contexts. However, since alignment across individuals is actually alignment of production and comprehension, an implicit corollary of alignment across interlocutors is the assumption that production and comprehension are also aligned within the individual, who is using both production and comprehension across the dialogue turns.

These models [i.e., the P-Chain Model and Pickering and Garrod’s (2004, 2013) Interactive Alignment model, and integrated theory of language production and comprehension] all suggest that production and comprehension are linked, in terms of the processes they draw upon or that they rely upon shared linguistic representations. The cross-modality structural priming studies discussed below show evidence of this link in the direction of comprehension to production, supporting the prediction of the P-Chain model (Dell and Chang, 2014). The current study adds to this literature by replicating comprehension to production priming, but also specifically testing whether there is evidence of the production-comprehension link in priming from production to comprehension. Testing this direction of priming will help to adjudicate between the P-Chain model, which does not predict priming in this direction, and Pickering and Garrod’s (2004, 2013) models. Moreover, for this priming task, we introduce a novel cross-modality structural priming paradigm using an online measure of comprehension, Event-Related brain Potentials. This temporally sensitive measure allows for an understanding of the time course and nature of processing elicited in the paradigm. More generally, examining cross-modality priming in both directions provides a more complete understanding of the relationship between the modalities and whether this relationship manifests differently depending on the target modality.

### Structural Priming

Structural, or syntactic, priming is a phenomenon in which the processing of a target item is facilitated by having recently encountered a similar item. Priming occurs for syntactic structures that have multiple alternative constructions, such as being able to describe a transitive event using either the active or passive voice. In production, priming occurs when an individual is more likely to use one construction (e.g., the passive voice) after having encountered that alternative rather than the other (i.e., the active voice). In comprehension, priming is found in facilitated comprehension of primed as compared to unprimed structures. Syntactic priming has been found in production (e.g., Bock, 1986; for a meta-analysis, see Mahowald et al., 2016) and comprehension (e.g., Branigan et al., 2005), in different paradigms (picture description, e.g., Melinger and Dobel, 2005; sentence completion, e.g., Scheepers, 2003), in different modalities (writing as well as speaking, e.g., Hartsuiker et al., 2008), in children (e.g., Van Beijsterveldt and Van Hell, 2009), with different methodologies (event-related potentials, ERPs, e.g., Ledoux et al., 2007; functional magnetic resonance imaging, fMRI, e.g., Segaert et al., 2013), both within- and across-languages in bilinguals (e.g., Loebell and Bock, 2003) and in code-switching in bilinguals (Kootstra et al., 2012), and in natural speech (corpus data, e.g., Gries, 2005; Jaeger and Snider, 2008; Torres Cacoullos and Travis, 2016).

Though most studies of structural priming within an individual examine priming in one modality, most often from production-to-production (e.g., Bock, 1986; for a review, see Mahowald et al., 2016), two studies have compared priming in production and comprehension (Bock et al., 2007; Tooley and Bock, 2014). Tooley and Bock (2014) compared within-modality comprehension-to-comprehension priming with within-modality production-to-production priming. Participants completed priming tasks of transitive (main clause vs. reduced relative clause) and dative sentences (prepositional object vs. double object alternation) with and without verb overlap between prime and target. For both priming tasks, each prime and target trial consisted of three steps: (1) an initial sentence that was read via rapid serial visual presentation (RSVP), (2) a digit memorization distractor task (to prevent sentence rehearsal), and (3) *either* a comprehension trial, in which participants read another sentence via self-paced reading and had to decide if this sentence matched the sentence from step 1, *or* a production trial, in which participants saw the word “Repeat,” had to repeat aloud the RSVP sentence presented in step 1, and had to decide if they had correctly produced the sentence verbatim or not. Comprehension-to-comprehension priming consisted of two successive comprehension trials, and the dependent measure was self-paced reading times for primed and unprimed sentences. Production-to-production priming consisted of two successive production trials, and the dependent measure was proportion of productions made in the primed structure, calculated as the number of sentences correctly recalled in the primed structure or switched to the priming sentence’s structure. Across both priming modalities, priming was found for both dative and transitive structures, and this priming was greater when there was lexical verb overlap. Importantly, the degree of priming was comparable in production-to-production and in comprehension-to-comprehension priming. From this within-modality structural priming, Tooley and Bock (2014) concluded that language processing in both modalities relies on similar structural mechanisms.

Finding priming from one modality to the other, in a cross-modality structural priming paradigm, would provide stronger evidence that production and comprehension share representations or processing mechanisms.

Bock et al. (2007) explicitly compared the effects of cross- modal comprehension-to-production priming to production-to-production priming across studies. Participants had to: (1) listen to a prime sentence; and (2) subsequently describe a picture to produce the target sentence for transitive (active vs. passive) and dative (prepositional object vs. double object) sentences. Significant cross-modality comprehension-to-production priming was found in terms of syntactic choice. This cross-modality priming was then compared to performance from Bock and Griffin (2000) where participants completed the same priming task, but between steps 1 and 2, repeated the prime sentence out loud. Thus, they: (1) listened to a prime sentence, (2) repeated that prime sentence aloud, and (3) then described a picture to produce the target sentence. Steps 2 and 3 created a production-to-production priming task. Priming was found within this production-to-production priming task in terms of syntactic choice, and the strength of the priming effect was statistically comparable across the Bock et al. (2007) and Bock and Griffin (2000) studies, suggesting that within-modality and cross-modality priming picked up on abstract syntactic representations that are shared across the modalities.

However, Tooley and Bock’s (2014) and Bock and Griffin’s (2000) production priming reveals a methodological quirk that is found in many production priming experiments: that the production priming actually also involved comprehension since step 1 of a trial consisted of reading a sentence via RSVP, often in both prime and target processing, making it more of a cross-modal priming task. Even Bock’s (1986) seminal study on structural priming in language production involved comprehension in the prime trial. That is, prime trials consisted of listening to an auditorily presented sentence and repeating that aloud. Target trials then consisted of describing a picture. Thus, this structural priming is actually a form of comprehension-production-production priming. Yet, in these studies, the inclusion of both modalities has not been expressly manipulated or overtly acknowledged, and as such, precludes conclusions about the influence of one modality on the other. While the comparison between Bock et al.’s (2007) comprehension-to-production priming and Bock and Griffin’s (2000) production-to-production priming relies on the subtraction of the intermediate production step to test whether two priming tasks produce similar results (and indeed they did), these studies were not designed to examine the effect of strictly comprehension processing on strictly production processing. They do, however, provide an excellent foundation from which to examine the influence of strictly production processing on strictly comprehension processing (and vice versa) in the current study.

Thus far, cross-modality structural priming from comprehension to production has been found (e.g., Bock et al., 2007) and therefore demonstrates some overlap in the underlying processes of the two modalities. There has been much less research on structural priming within comprehension (see reviews by Pickering and Ferreira, 2008; Branigan and Pickering, 2017), and, to our knowledge, only two studies have examined cross-modality structural priming from production to comprehension (Branigan et al., 2005; Segaert et al., 2012). However, only one (Segaert et al., 2012) compared priming in both directions, but neither used a temporally sensitive online measure of comprehension.

Branigan et al. (2005) studied ambiguity resolution in prepositional phrase attachment, and whether structural priming could affect sentence interpretation focusing on comprehension-to-comprehension priming. Participants completed prime trials in which they read an ambiguous expression (e.g., “*The waitress prodding the clown with the umbrella*”) and saw two pictures, one corresponding to either high (i.e., waitress with the umbrella) or low attachment (i.e., the clown with the umbrella) and the other to neither, thus forcing one type of ambiguity resolution. On target trials, participants again read a sentence and saw two pictures, but this time one picture corresponded to high attachment and one to low attachment. Participants’ picture choice served as the measure of ambiguity resolution. Priming was found when the verb was repeated across prime and target trials but not when different verbs were used, in that participants more often chose the high attachment interpretation following a prime trial which had only a high attachment picture option. This comprehension-only priming was then compared with production-to-comprehension priming. Here, production primes consisted of participants reading a verb and using that verb to describe a picture that was created to induce either a high- or low-attachment phrase. Cross-modality priming was again found, and was comparable in magnitude to comprehension-only priming. However, this study did not include a comprehension-to-production priming direction for comparison, and used an off-line target task that does not allow for real-time analysis of processing.

Segaert et al. (2012) directly compared within-modality (production-to-production and comprehension-to-comprehension) priming with cross-modality (production-to-comprehension and comprehension-to-production) priming of active and passive sentences in an fMRI task in which participants listened to sentences and described pictures. Specifically, for comprehension trials, participants viewed a greyscale picture and listened to an accompanying sentence. For 10% of comprehension trials (including filler trials), there was a mismatch between the picture and the sentence, and participants had to respond to the mismatch. For production trials, participants saw a color-coded image with a verb presented and had to describe the picture, naming the actor colored in green first, followed by the actor in red. The color-coding forced either an active or passive sentence structure. Priming effects were evidenced by repetition suppression for primed versus unprimed targets. Repetition suppression is an effect wherein a neuronal population responds less strongly when a stimulus has been repeated, as compared to the first presentation. That is, the neuronal population supporting production processing fires less strongly on a target trial if it has just been activated on a production prime trial. Here, Segaert et al. (2012) asked whether production and comprehension relied on the same neuronal population and if this repetition suppression would be found between a comprehension prime trial and a production target trial (or vice versa). This priming effect, the difference between primed and unprimed active and passive sentences, was found in the same brain regions for within- and cross-modality priming (left middle temporal gyrus, left inferior frontal gyrus, bilateral supplementary motor area), indicating that production and comprehension of transitive structures rely on the same neuronal populations. These data provide evidence that production and comprehension rely on similar neural substrates within an individual. However, this study cannot speak to the relationship between production and comprehension in online processing. While fMRI provides excellent fine-grained spatial information, it cannot provide equivalent temporal information sensitive to ease of processing in real-time. Additionally, in the Segaert et al. (2012) study participants were always cued as to which syntactic construction to use in production. Such fixed productions do not accurately reflect natural language use. Use of ERPs in the current study allows for further understanding of the relationship between the modalities during online processing.

### Current Study

Recent theories (Pickering and Garrod, 2004, 2013; MacDonald, 2013; Dell and Chang, 2014) have suggested a link between the processes underlying production and comprehension, though differ on their interpretation of the link from production to comprehension. Empirical support for this idea has come from structural priming, and specifically cross-modality structural priming from comprehension to production. The present study uniquely studies cross-modality structural priming in both directions, from production into comprehension as well as from comprehension into production, combining behavioral, and Event-Related Potential measures. The inclusion of the *Production-to-Comprehension* priming task extends the small literature on comprehension-to-comprehension priming (compared to the wealth of studies on priming into production; Pickering and Ferreira, 2008; Branigan and Pickering, 2017), provides a test of the theories of production and comprehension discussed above [the P-Chain Model, Dell and Chang, 2014; Interactive Alignment model, Pickering and Garrod’s (2013)]. On a methodological note, the scope of comprehension-to-production priming is limited to the study of syntactic constructions that have two alternatives with the same meaning, whereas production-to-comprehension priming does not have such a restriction as priming is measured by the facilitation of target processing depending on the preceding context. Thus, the paradigm developed here can be adapted to the study of syntactic constructions not yet explored in traditional structural priming studies to investigate whether priming depends on the specific construction or reflects a general principle of production and comprehension. Moreover, the present study is the first to use a temporally sensitive measure for online comprehension in this cross-modal direction. The use of ERPs in comprehension priming adds insight into the online, neurocognitive mechanisms underlying cross-modality priming (to be discussed in more detail below). In comparison to behavioral measures, ERPs provide a multidimensional window that not only delineates whether there is a numerical benefit of the prime on target process, but also the nature of that processing.

To examine whether the nature of the relationship between comprehension and production depends on the direction of priming, we investigated priming from production to comprehension and vice versa. English monolinguals completed both a *Comprehension-to-Production* and a *Production-to-Comprehension* structural priming task focusing on the active/passive alternation (*The girl was helping the boy* vs. *The girl was helped by the boy*). For *Comprehension-to-Production* priming, the dependent measures were syntactic choice, as well as two measures of speed of response: response time (RT) and average syllable duration (Gahl and Garnsey, 2004; Quené, 2008; Fricke et al., 2016). Given previous findings of cross-modality priming into production (e.g., Branigan et al., 2005), we expected to find significant priming. Specifically, for syntactic choice, we expected a higher proportion of passives produced following passive, rather than active, primes. This priming pattern is expected for the production of passive sentences, rather than for the production of active sentences due to the inverse frequency effect (Chang et al., 2006; Jaeger and Snider, 2008), the phenomenon that stronger priming is found for the less frequent structure. For speed of response measures (RT and average syllable duration), the small literature (Corley and Scheepers, 2002; Segaert et al., 2011, see Pickering and Ferreira, 2008) that measured response latency in structural priming has found a pattern opposite to that of syntactic choice. For example, in Segaert et al.’s (2011) study of active/passive production-to-production picture description priming, syntactic choice priming was found in terms of more passive responses produced following passive, rather than active primes, while no response latency priming was found on these passive productions. The authors conducted a follow-up study in which some participants were given a training block prior to the priming task in which they encountered 90% passive sentences and 10% active sentences to boost the relative frequency of passive sentences relative to general language use. Following this training block, priming was found for passive sentences in both syntactic choice and RT measures. The authors interpret these effects as indicating that unlike syntactic choice, response latency priming is found for the more frequent structure. Therefore, given these results, for response latency measures, we examined the effect of priming on both passive and active productions and expected to find little or no latency priming on passive productions, but significant priming on active productions.

For the novel *Production-to-Comprehension* priming paradigm, the dependent measure was ERPs. If the link between production and comprehension is bidirectional, significant cross-modality priming should also be found into comprehension. ERPs reflect a direct measure of the electrical activity produced by the brain and provide a fine-grained measure with temporal resolution at the level of milliseconds that reveal covert language processing before a behavioral response is possible. We specifically focused on the N400 and P600 components. The N400 is a negative component peaking between 300 and 500 ms over centro-parietal sites that typically indexes lexico-semantic access and integration (Kutas and Hillyard, 1980). The P600 is a positive component arising between 500 and 900 ms over posterior sites that typically indexes syntactic processing (e.g., Osterhout and Holcomb, 1992; Hahne and Friederici, 1999). Previous studies of comprehension structural priming using ERPs have typically found a reduction in the P600 effect to indicate facilitated processing of primed syntactic structures (Ledoux et al., 2007; Tooley et al., 2009, 2013; Boudewyn et al., 2014; for a review, see Tooley and Traxler, 2010). Earlier positivities (Ledoux et al., 2007) and N400 effects to repeated verbs (Ledoux et al., 2007; Tooley et al., 2009) have also been found. The previous structural priming studies have all examined visually presented comprehension-to-comprehension priming, and have mostly focused on the influence of repeated words across primes and targets. The current study examines auditorily presented production-to-comprehension priming, and therefore may show (slightly) different ERP responses [see, e.g., Litcofsky and Van Hell (2017) and Fernandez et al. (2019) for different ERP and time-frequency analysis responses to visually- and auditorily presented codeswitched sentences, respectively]. We examined priming at the main verb in passive targets at both early and late time windows. We expected a reduction in the ERP effect (possibly a P600) for primed passive targets (preceded by passive primes) as compared to unprimed passive targets (preceded by active primes; Tooley and Traxler, 2010).

## Materials and Methods

### Participants

Twenty-four English monolinguals were tested for this study. Data from three participants were discarded: one due to excessive blink artifact in the EEG signal, and two who, after testing, were found to not meet inclusion criteria. Data from the remaining 21 participants (17 female) were analyzed (age: *M* = 19.09, *SD* = 1.0). All participants were native speakers of English who had limited experience with foreign languages (self-rated proficiency in second language out of 10: *M* = 2.21, *SD* = 1.45). Foreign language experience was assessed via an in-house questionnaire. All participants were right-handed and reported normal or corrected-to-normal vision, and no brain trauma. They were recruited from the Penn State Psychology Subject Pool and received course credit for their participation. All participants provided informed consent before the experiment.

### Materials

The critical stimuli consisted of 400 sentences in the active/passive construction. This structure has shown significant unimodal priming (e.g., Bock, 1986) and cross-modality production/comprehension priming (Segaert et al., 2012). Critical sentences were presented and elicited in the past tense (Active: *The girl was helping the boy*; Passive: *The girl was helped by the boy*) so that both active and passive sentences have the same number of morphemes in the verb phrase and the verb phrase begins with the same letter “w”, which is relevant for the reliability of the online production measures (RTs and syllable duration) and the ERP measurements (Luck, 2014). Materials were divided into two priming tasks: *Comprehension-to-Production* and *Production-to-Comprehension*.

Critical sentences for both priming tasks were created from a set of twenty animate actors and forty transitive verbs (see Appendix). Each sentence contained two nouns (one agent and one patient) and one verb. Sentences never contained repeated nouns, and across sentences, while individual nouns and verbs appeared across multiple items, the set of two nouns and a verb (e.g., “girl,” “boy,” “help” in “*The girl was helping the boy*”) never appeared more than once. Each prime/target pair contained sentences in which the verb was repeated (given that priming in comprehension often relies on verb overlap, see Branigan et al., 2005; Tooley and Traxler, 2010), but the actors were different. Verbs varied across different prime/target pairs. Different sentences were used for active and passive prime/target pairs. A total of 400 experimental sentences were created for both priming tasks.

To mask the critical manipulation, filler sentences were created, including locative and intransitive sentences. There were twice the number of fillers as experimental stimuli in each priming task. 600 locatives were created from a set of 100 inanimate objects and 14 prepositions to make sentences such as “*The iron was near the basket*.” 200 intransitives were created from a set of 50 animal nouns and 8 intransitive verbs to make sentences such as “*The dolphin was playing*.”

### Procedure

#### Comprehension-to-Production Priming

For the *Comprehension-to-Production priming* task, participants first listened to a prime sentence and then produced a target sentence by describing a picture (see Table 1 for a schematic).

**Table 1:** Depiction of priming for comprehension-to-production and production-to-comprehension tasks.

Priming Direction	Prime	Target
Comprehension-to-Production
	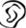	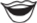
Production-to-Comprehension
	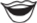	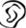


*Ear, Listening for comprehension; Mouth, Speaking for production.*

There were 80 prime/target pairs consisting of 80 pre-recorded auditorily presented prime sentences and 80 target images to be described by the participant. Images were taken from the International Picture-Naming Project (Szekely et al., 2004). Of the prime sentences, 40 were active and 40 were passive, and participants listened for comprehension. All sentences were pre-recorded by a native English-speaking female. In Praat (Boersma and Weenink, 2014), sentence recordings were normalized to 70 dB, sampled at a rate of 44,100 Hz, and 50 ms of silence was added to the beginning and end of the sentences to act as a buffer.

**FIGURE 1:**
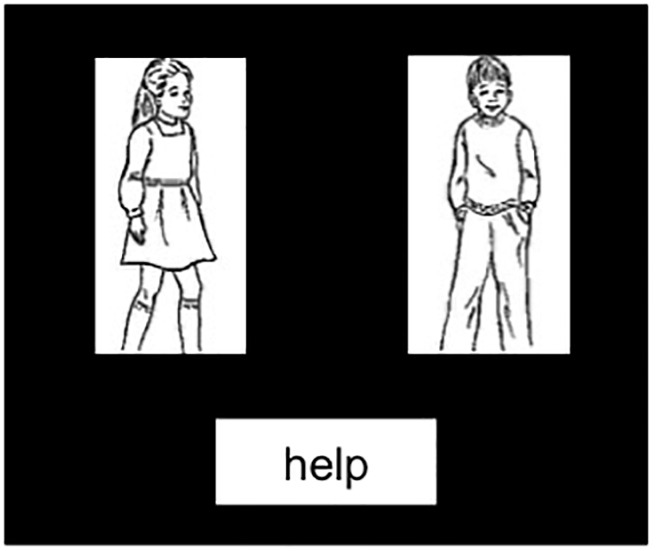
Example production target image. Participants were instructed to name from left to right so descriptions could be active, *The girl was helping the boy*, or passive, *The girl was helped by the boy.*

The target images (see Figure 1) were black screens with two black-and-white line drawings on a white background of animate actors (an agent and patient), with a verb printed below (e.g., *help*; text in black on white box). Participants were instructed to produce a sentence involving both actors, named from left-to-right and the verb in either the active (e.g., *The girl was helping the boy*) or passive (e.g., *The girl was helped by the boy*) voice construction. 240 locative (half production, half comprehension) and 80 intransitive (half production, half comprehension) filler items were presented similarly to critical sentences: for locatives, a preposition was printed on the target image (e.g., *behind*), and for intransitives, there was only one image of an actor with the other white box remaining blank for visual consistency across items.

**FIGURE 2:**
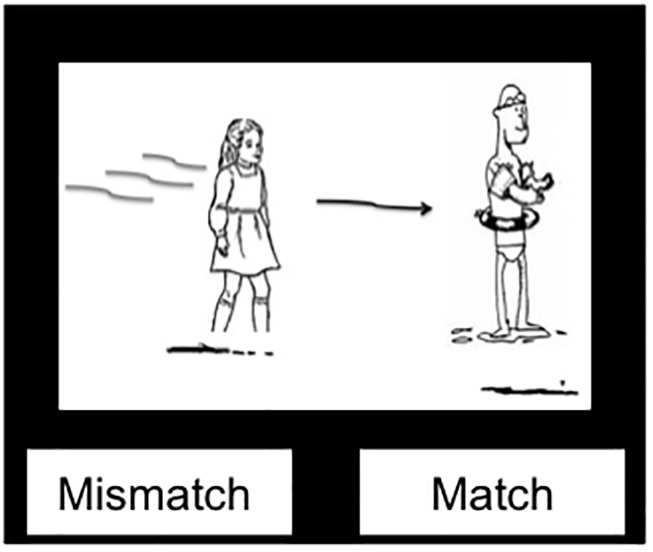
Example picture verification image.

To ensure that participants were engaged in the comprehension task, a picture verification task followed half of the comprehension primes in which a black-and-white picture along with a “Mismatch/Match” prompt appeared. Participants had to decide whether the picture matched the just-heard auditory sentence (Hartsuiker et al., 2004; Segaert et al., 2012; see Figure 2). Half of the picture verification trials were “match” trials and half “mismatch” (with a different actor, verb or preposition, or assignment of actors to agent/patient roles than in the auditory sentence). Note that the inclusion of these picture verification questions on half of the comprehension trials adds slightly to the lag between prime and target. However, this is minimal compared to the presence of intervening filler trials, and the picture verification questions were split evenly across trial types.

Practice trials for the *Comprehension-to-Production* priming task consisted of 20 prime/target pairs of critical sentences (5 of which were also used in the practice trials for the *Production-to-Comprehension* task), 26 locatives, and 14 intransitives, and were presented in the same manner as the experimental stimuli. Oral feedback was given to participants by the experimenter regarding how to name the pictures (e.g., naming from left-to-right, or to use “*was* helping” or “*was* helped by” rather than “*helps*” or “*is* helped by”), but not whether to use an active or passive construction. The timing of the trials was as follows. Before all trials, a self-paced “Ready?” screen appeared. Then, for comprehension trials, a white fixation cross on a black background was presented for 300 ms prior to the sentence, signaling that an auditory sentence was next. The sentence was then presented auditorily through insert earphones (Etymotic Research, Elk Grove Village, IL, United States) while the fixation cross remained on the screen, followed by a 500 ms blank screen. If the trial contained a picture verification question, there was a 100 ms blank screen followed by the picture, which remained on the screen until the participant made their choice by pushing one of two buttons, followed by another 100 ms blank screen. For production trials, a white fixation box on a black background was presented for 300 ms, signaling the upcoming trial was a picture description, followed by a 100 ms blank screen and then the picture was presented. The picture remained on the screen until the participant began their description into a recording microphone, and remained on the screen for an additional 1500 ms while they produced their description. A 500 ms blank screen followed the description. There was a 100 ms blank screen between all trials.

#### Production-to-Comprehension Priming

**FIGURE 3:**
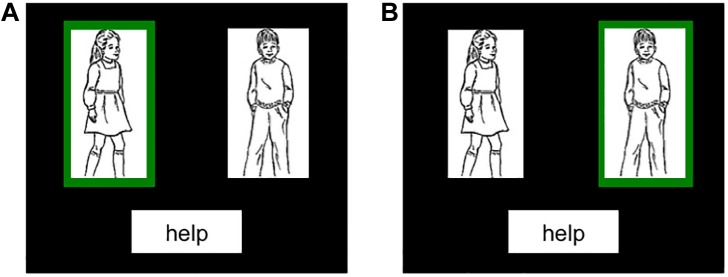
Example production prime images. Participants were instructed to name from left to right and that the person in green did the action to the other person. The descriptions would be **(A)** active, *The girl was helping the boy*, **(B)** passive, *The girl was helped by the boy*.

For the *Production-to-Comprehension* priming task, the participants first produced a prime sentence by describing a picture and then subsequently listened to a pre-recorded target sentence (see Table 1). Like production targets described above, production primes consisted of two actors with a verb written underneath, but here one actor was surrounded by a green border (see Figure 3). Participants were again instructed to name the actors left-to-right, but here additionally were told that the actor outlined in green did the action to the other actor. For example, in a picture with a girl on the left and a boy on the right, a girl in green would yield “*The girl was helping the boy*” and a boy in green would yield “*The girl was helped by the boy*.”

Forty of these pictures yielded active primes and 40 passive primes. The 80 targets consisted of participants listening to pre-recorded auditorily presented sentences (see above for details). These targets were all in the passive construction, for two reasons: (1) to reduce the number of trials needed in the experiment (including active targets would require two additional conditions: active-active primed pairs and passive-active unprimed pairs); (2) passives generally show stronger priming effects than actives (Bock, 1986; Hartsuiker et al., 2004; Segaert et al., 2012). However, to maintain a balance between active and passive sentences, an additional 40 active primes and 40 active targets were included following the same constraints as above (increasing the number of trials, but not requiring the additional condition of passive prime – active target). Thus, there were a total of 120 prime/target transitive sentences. 360 locative (half production, half comprehension) and 120 intransitive (half production, half comprehension) filler prime sentences were presented similarly to the critical sentences, but did not include the green square manipulation. Like for comprehension-to-production priming, there was a picture verification task on half of the comprehension sentences.

Practice for the *Production-to-Comprehension* priming task consisted of 20 prime/target pairs of critical sentences (5 of which were also used in the practice for *Comprehension-to-Production*), 26 locatives, and 14 intransitives, and was presented in the same manner as the experimental stimuli. Feedback was given to participants regarding how to name the pictures (e.g., naming from left-to-right, that the person in green did the action to the other person, to use “*was* helping” or “*was* helped by” rather than “*helps*” or “*is* helped by”), but not whether to use an active or passive construction.

Timing parameters of the comprehension and production trials were the same as described above for the Comprehension-to-Production priming task.

#### Experimental Session

The two priming tasks were conducted across two experimental sessions, separated by approximately 1 week (*M* = 6.67 days, *SD* = 1.49). There were two experimental lists. In the first list, the *Comprehension-to-Production* task occurred on testing day 1, and the *Production-to-Comprehension* task on testing day 2. In the second list, the order of the priming tasks was reversed. Moreover, the order of the blocks within each task was counterbalanced. For the *Comprehension-to-Production* task, critical and filler stimuli were intermixed into 3 blocks of 160 items each. For the *Production-to-Comprehension* task, critical and filler stimuli were intermixed into 4 blocks of 180 items each. For both tasks, critical active/passive primes and targets were separated by 0, 1, or 2 fillers, evenly distributed. Both priming tasks were audio-recorded so that production descriptions could be coded both on-line (via a playback function in the recorder) and off-line by a native speaker.

To quantify participants’ language proficiency and executive functions, they completed a language history questionnaire (collecting demographic information, self-report measures on language acquisition, exposure, proficiency, use, literacy, and attitudes), and a series of individual difference measures: a picture naming language proficiency task (the Boston Naming task, BNT; Kaplan et al., 2001), a working memory task (the automated Operation Span task; Conway et al., 2005; Unsworth et al., 2005; Redick et al., 2012), an inhibitory control task (the AX-CPT, Morales et al., 2013), and a task of implicit bias regarding foreign-accented speakers of English (the Implicit Association Task, IAT; Greenwald et al., 1998; Lane et al., 2007)^1^. The language history questionnaire was completed during EEG setup on testing day 1. The operation span, picture naming, and implicit association tasks were completed following the *Comprehension-to-Production* task in one testing session and the AX-CPT task was completed following the *Production-to-Comprehension* task in the other testing session.

### EEG Recording and Preprocessing

For both priming tasks, EEG was recorded throughout the task but only analyzed for comprehension target trials (in *Production-to-Comprehension* priming), because ERPs recorded during ongoing speech are contaminated by movement artifacts. EEG was recorded in both priming tasks to ensure that the experimental sessions appeared similar to participants across priming tasks to not influence priming. Participants were seated in a comfortable chair about three feet from the computer in a sound-attenuated darkened chamber. An elastic cap (Brain Products ActiCap, Germany) with 31 active Ag/AgCl electrodes was placed on the participant’s head. Electrode locations consisted of five sites along the midline (Fz, FCz, Cz, Pz, Oz) and 26 lateral electrodes (FP1/2, F7/8, F3/4, FC5/6, FC1/2, T7/8, C3/4, CP5/6, CP1/2, P7/8, P3/4, O1/2, PO9/10). In order to monitor vertical eye movements/blinks, bipolar recordings were made above and below the left eye, and the outer canthus of each eye. Electrodes were referenced to a vertex reference (electrode FCz) and re-referenced offline to an average of the left and right mastoids. The electroencephalogram (EEG) was amplified by a NeuroScan SynampsRT amplifier using a 0.05–100 hz bandpass filter and continuously sampled at a rate of 500 hz. Electrode impedances were kept below 10 kΩ.

Preprocessing and measurement of the ERP data was done in ERPlab (Lopez-Calderon and Luck, 2014). An off-line 30 Hz low-pass filter was applied. For each participant, separate ERPs were averaged off-line at each electrode site for each experimental condition, relative to a 200 ms prestimulus baseline. Target trials in which participants had incorrectly produced the prime sentence (e.g., when the green box was surrounding the first person indicating an active sentence but the participant produced a passive, or vice versa) were excluded for all target words (active prime: 2.9%, passive prime: 2.9%). Additionally, target words contaminated with eye artifact, but when the prime sentence was correctly produced, were not included (active prime: 15.91%, passive prime: 15.31%; “by”: active prime: 13.94%, passive prime: 12.95%; second noun: active prime: 15.30%, passive prime: 12.60%). Note that while these rates of rejection appear high, analyses were conducted on a sufficient number of trials per condition (across conditions and target words: Range: 31.62–33.95, *M* = 32.91, *SD* = 0.82).

### Data Analysis

For *Comprehension-to-Production* priming, three dependent measures were of interest for the critical target descriptions. The first was Syntactic Choice. Descriptions were coded as “active,” “passive,” or “other” by two trained individuals. In the rare cases of disagreement, the first author made the final call. The dependent measure for syntactic choice was the proportion of passive sentences produced, out of both active and passive sentence productions. Priming would be shown by a higher proportion of passive descriptions following passive primes as compared to passive descriptions following active primes. For more sensitive measures of production, we also used two measures of speed of response: log of the RT, measured as the time between onset of the picture and onset of the description (e.g., Corley and Scheepers, 2002); and Average Syllable Duration (Gahl and Garnsey, 2004; Quené, 2008; Fricke et al., 2016), which, to our knowledge, has not yet been applied to structural priming paradigms. Priming would be shown by faster RTs and shorter average syllable durations for primed, as compared to unprimed productions. For the two RT measures, both active and passive productions were analyzed. Outlier removal for RTs included an absolute cutoff of 300 ms and a relative cutoff of 2.5 standard deviations above and below the by-subject and by-condition means. Outlier removal of average syllable duration consisted of a relative cutoff of 2.5 standard deviations above and below the by-subject and by-condition means. The predictor variables were Prime (active, passive) as well as Order^2^ (1–40, the number of the passive or active sentences comprehended at that trial, depending on the analysis). Order was included as a main effect to examine any cumulative priming (Chang et al., 2006; Jaeger and Snider, 2008), and as an interaction with Prime to examine how priming may change over the course of the experiment.

Syntactic choice was analyzed via mixed effects logistic regression models and RT and Average Syllable Duration were analyzed via mixed effects regression models (Baayen et al., 2008) using the lme4 package Bates et al. (2015) in version 3.4.0 of R (R Development Core Team, 2008). Random intercepts for participants and items, as well as by-participant random slopes for Prime and by-item random slopes for Order were included. For each model, the random effects structure reflected the maximal structure supported by the design (Barr et al., 2013). That is, all models started with a full random effects structure. Then, random slopes were removed due to non-convergence and random slopes correlated above 0.95 were removed to avoid over-fitting. *P*-values were calculated via model comparison using chi-square tests. Each analysis included two fixed main effects: Prime (contrast coded, baseline or reference category set as active prime, or the unprimed condition) and Order (continuous variable, centered to its mean), as well as the interaction of Prime and Order.

For *Production-to-Comprehension* priming, target passive comprehensions were assessed via EEG. EEG was time-locked to the onset of the main verb in the passive targets (e.g., “helped” in *The girl was helped by the boy*; see Supplementary Material for analyses of the auxiliary verb “*was*,” the word “*by*,” and the second noun “*boy*”). Analysis of the critical word was conducted on mean amplitudes with a baseline of 200 ms pre-stimulus activity. In accordance with previous studies and visual inspection of the data, two time windows were analyzed, corresponding to the epochs of the N400 and P600: 300–500 ms and 500–900 ms post word onset.

Two repeated measures analyses of variance (ANOVA) were performed to examine the scalp distribution of the ERP effect. One ANOVA focused on midline electrodes and included a factor of electrode group (Fz, Cz, Pz). The second ANOVA included a factor of anteriority (anterior, posterior) and laterality (right, left hemisphere). For these factors, electrodes were grouped into regions of interest: right frontal (“RF”: F4, F8, FC2, FC6); left frontal (“LF”: F3, F7, FC1, FC5); right posterior (“RP”: CP2, CP6, P4, P8); left posterior (“LP”: CP1, CP5, P3, P7). A Greenhouse–Geisser correction was applied to analyses with more than one degree of freedom in the numerator. Significant interactions were examined further with simple effects tests and planned comparisons. Factors of prime type (active, passive) as well as of experimental half (1st half, 2nd half) were included to investigate whether the priming effect changed throughout the course of the experiment.

Finally, to examine whether there is an explicit link between the two tasks, we correlated the magnitude of the significant priming effects observed in each task. Priming effects were calculated as the difference in response measures between the primed and unprimed condition.

## Results

### Comprehension-to-Production

#### Syntactic Choice

**FIGURE 4:**
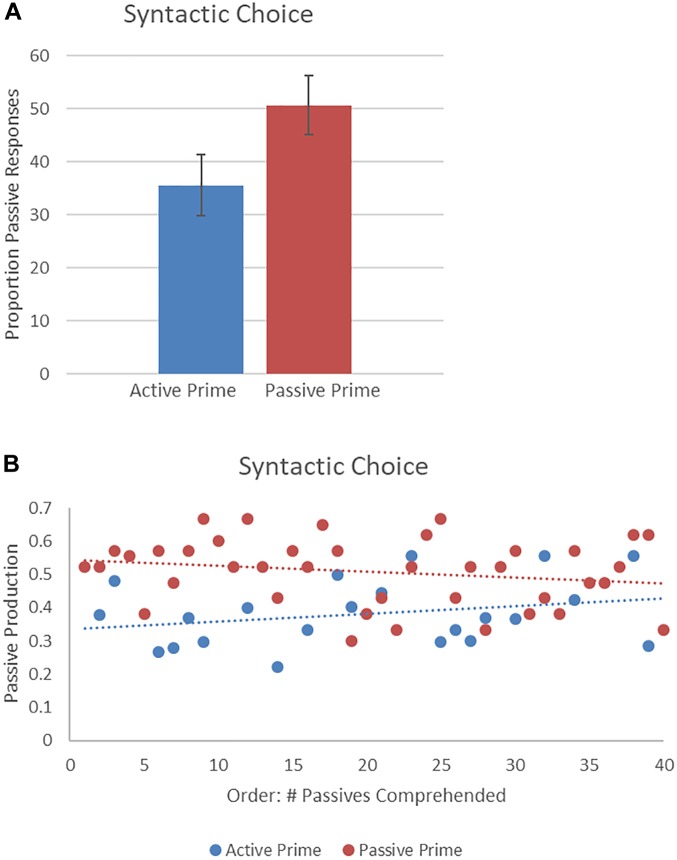
**(A)** Effect of prime (active: blue, passive: red) on syntactic choice, shown as proportion of passives produced. Error bars represent standard error. **(B)** Effect of prime (active: blue, passive: red) on passives produced as a function of the number of passives comprehended.

Figure 4 presents descriptive syntactic choice results showing the proportion of passives produced. The final random effect structure included random intercepts for participants and items, and correlated by-participant random slopes for prime. Table 2 presents a summary of the model. There was a significant effect of prime, such that a higher proportion of passive descriptions were produced following passive prime sentences, rather than active prime sentences. There was no effect of order and no interaction of prime and order. Thus, the higher likelihood of producing a passive sentence following a passive prime, rather than an active sentence, remained the same throughout the experiment.

**Table 2:** Summary of the mixed effects model for syntactic choice (comprehension-to-production priming).

Predictor	Parameter estimates		Δ(-2λ) test	
		
Fixed Effects	Estimate	*SE*	χ^2^	*p*
(Intercept)	-0.543	0.353		
Prime	0.968	0.321	9.435^a^	<0.01
Order	-0.002	0.005	1.647	0.439
Prime × Order	-0.013	0.011	1.417	0.234


*^a^Likelihood ratio tests for main effects are based on omitting the main effect as well as the interaction.*

#### Response Time

**FIGURE 5:**
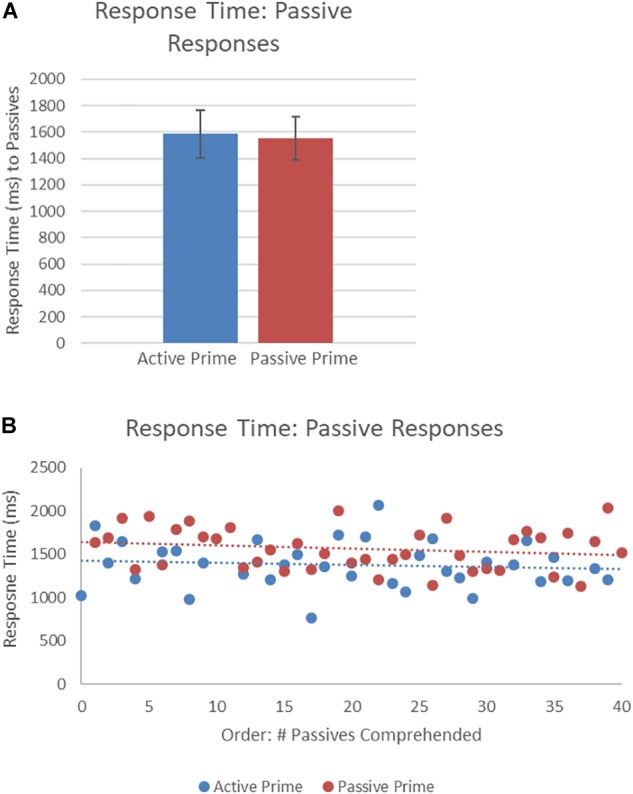
**(A)** Effect of prime (active: blue, passive: red) on response time (RT) to passive targets. Error bars represent standard error. **(B)** Effect of prime (active: blue, passive: red) on RT to passive targets as a function of the number of passives comprehended. Note that raw RTs are depicted though logRT was used for statistical analysis.

Figure 5 presents the RTs of passive structures as a function of prime type. The final random effect structure for passive responses included random intercepts for participants and items, and correlated by-participant random slopes for prime. Table 3 presents a summary of the model. There were no significant effects of prime or the interaction of prime and order, but there was a significant effect of order. Thus, passive responses were produced more quickly when they had comprehended more passive sentences, later in the experiment.

**Table 3:** Summary of the mixed effects model for response time (RT) for passive responses (comprehension-to-production priming).

Predictor	Parameter estimates			Δ(-2λ) test	
		
Fixed Effects	Estimate	*SE*	*t*-value	χ^2^	*p*
(Intercept)	7.220	0.087	83.491		
Prime	0.008	0.025	0.033	0.218^a^	897
Order	0.003	<0.001	-3.697	14.430	<0.001
Prime × Order	<-0.001	0.002	-0.306	0.096	0.756


*^*a*^Likelihood ratio tests for main effects are based on omitting the main effect as well as the interaction.*

**FIGURE 6:**
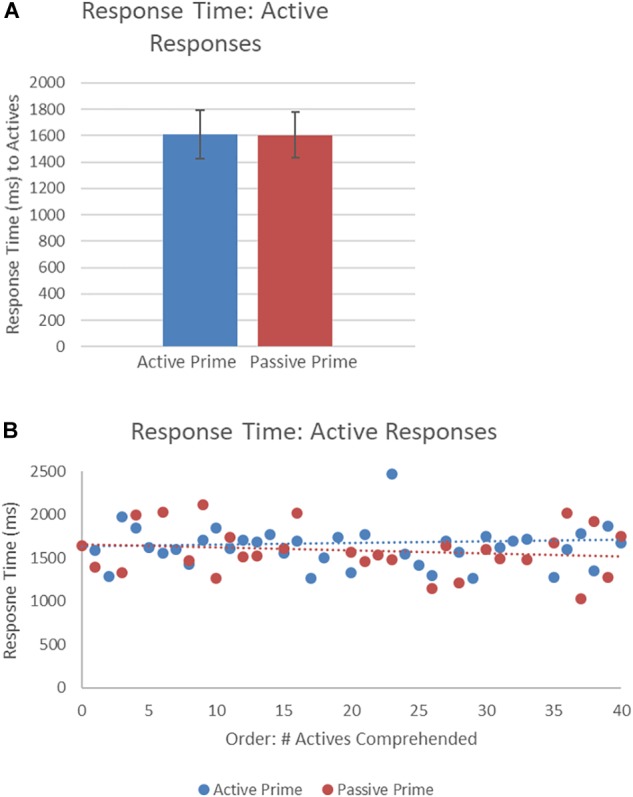
**(A)** Effect of prime (active: blue, passive: red) on RT to active targets. Error bars represent standard error. **(B)** Effect of prime (active: blue, passive: red) on RT to passive targets as a function of the number of actives comprehended. Note that raw RTs are depicted though logRT was used for statistical analysis.

Figure 6 presents the RTs of active structures as a function of prime type. The final random effect structure for active responses included random intercepts for participants and items, and correlated by-participant random slopes for prime. Table 4 presents a summary of the model. There was only a significant effect of order, indicating that, similar to passive responses, active responses were produced more quickly when they had comprehended more passive sentences, later in the experiment.

**Table 4:** Summary of the mixed effects model for RT for active responses (comprehension-to-production priming).

Predictor	Parameter estimates			Δ(-2λ) test	
		
Fixed Effects	Estimate	*SE*	*t*-value	χ^2^	*p*
(Intercept)	7.225	0.087	82.803		
Prime	0.041	0.026	1.556	2.443^a^	0.295
Order	-0.002	<0.001	-3.204	10.454	<0.01
Prime × Order	<-0.001	0.002	-0.033	0.002	0.966


*^*a*^Likelihood ratio tests for main effects are based on omitting the main effect as well as the interaction.*

#### Average Syllable Duration

**FIGURE 7:**
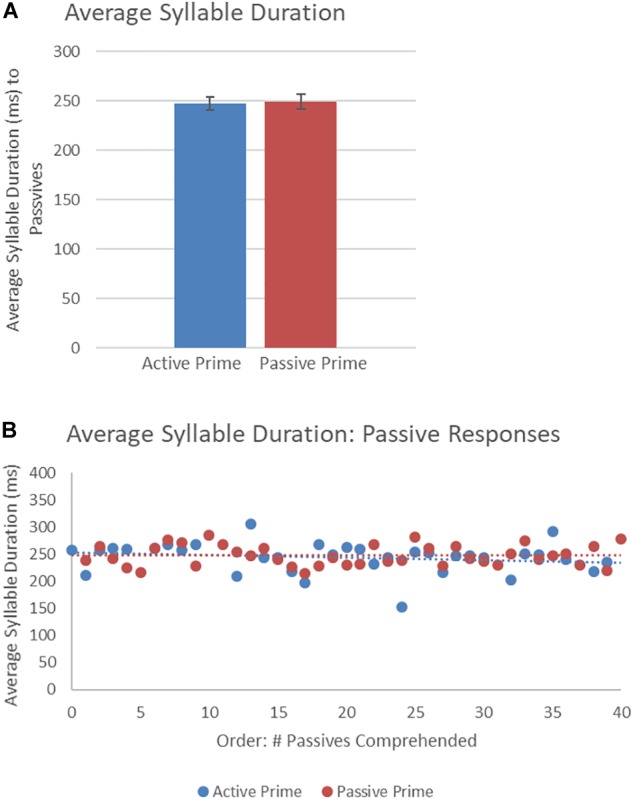
**(A)** Effect of prime (active: blue, passive: red) on average syllable duration of passive targets. Error bars represent standard error. **(B)** Effect of prime (active: blue, passive: red) on average syllable duration of passive targets as a function of the number of passives comprehended.

Figure 7 presents the average syllable duration of passive structures as a function of prime type. The final random effect structure for passive responses included random intercepts for participants and items, and correlated by-participant random slopes for prime. Table 5 presents a summary of the model. There were no significant effects of prime, order, or the interaction. Thus, no significant priming effects were detectable in the average syllable duration measure for passive responses.

**Table 5:** Summary of the mixed effects model for average syllable duration for passive responses (comprehension-to-production priming).

Predictor	Parameter estimates			Δ(-2λ) test	
		
Fixed Effects	Estimate	*SE*	*t*-value	χ^2^	*p*
(Intercept)	246.912	7.335	33.664		
Prime	-4.145	6.189	-0.670	1.451^a^	0.484
Order	-0.114	0.120	-0.946	1.567	0.457
Prime × Order	0.236	0.239	0.990	0.963	0.326


*^*a*^Likelihood ratio tests for main effects are based on omitting the main effect as well as the interaction.*

**FIGURE 8:**
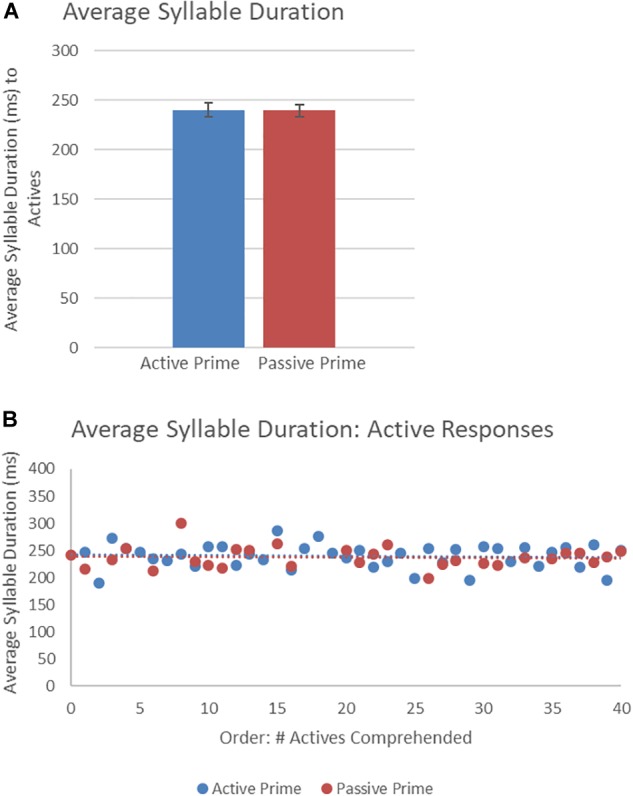
**(A)** Effect of prime (active: blue, passive: red) on average syllable duration of active targets. Error bars represent standard error. **(B)** Effect of prime (active: blue, passive: red) on average syllable duration of passive targets as a function of the number of actives comprehended.

Figure 8 presents the average syllable duration of active structures as a function of prime type. The final random effect structure for active responses included random intercepts for participants and items. Table 6 presents a summary of the model. There were no significant effects of prime, order, or the interaction. Thus, no significant priming effects were detectable in the average syllable duration measure for active responses.

**Table 6:** Summary of the mixed effects model for average syllable duration for active responses (comprehension-to-production priming).

Predictor	Parameter estimates			Δ(-2λ) test	
		
Fixed Effects	Estimate	*SE*	*t*-value	χ^2^	*p*
(Intercept)	236.976	6.953	34.080		
Prime	-1.989	4.549	-0.437	1.405^a^	0.496
Order	-0.044	0.126	-0.345	1.498	0.473
Prime × Order	0.273	0.249	1.098	1.218	0.270


*^*a*^Likelihood ratio tests for main effects are based on omitting the main effect as well as the interaction.*

### Production-to-Comprehension

**FIGURE 9:**
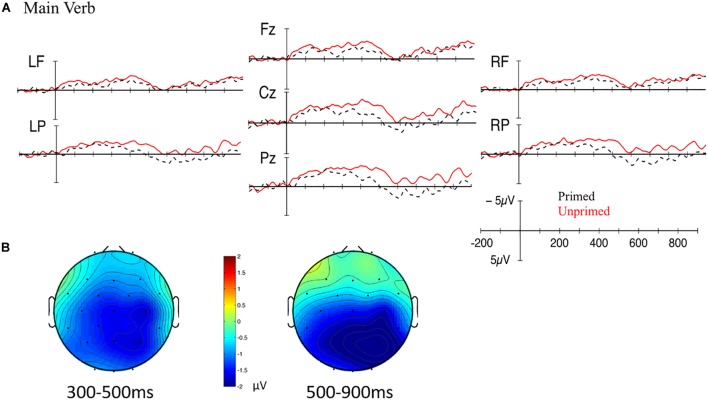
**(A)** Grand mean waveforms for the main verb for primed (passive prime – passive target; black dotted line) and unprimed (active prime – passive target; red solid line) conditions. Onset of the main verb is indicated by the vertical bar. The calibration plot shows amplitude is plotted on the *y*-axis (negative plotted up). Time is plotted on the *x*-axis; each tick mark indicates 100 ms. LF, left frontal; RF, right frontal; LP, left posterior; RP, right posterior. **(B)** Scalp topography maps showing the mean difference between unprimed and primed conditions between 300 and 500 ms (left) and 500–900 ms (right).

Grand mean waveforms and scalp plots for the main verb for primed (passive prime – passive target) and unprimed (active prime – passive target) conditions are plotted in Figure 9. Visually, there is a widespread extended negativity, which was confirmed by statistical analyses. In the 300–500 ms time window, there was a main effect of Prime such that the ERP waveform for primed sentences was less negative-going than that for unprimed sentences [midline: *F*(1,20) = 10.540, *p* = 0.004; lateral: *F*(1,20) = 6.698, *p* = 0.018]. There was a significant interaction between Prime, Half, Hemisphere, and Anteriority [lateral: *F*(1,20) = 9.826, *p* = 0.005], but follow-up analyses revealed no significant effects (all *p*s > 0.27). There was an effect of Half such that the ERPs were more negative-going during the second half than the first half of the experiment [midline: *F*(1,20) = 5.690, *p* = 0.027; lateral: *n.s.*], but this did not interact with the priming manipulation.

In the 500–900 ms time window, there was a main effect of Prime such that primed sentences were less negative-going than unprimed sentences [midline: *F*(1,20) = 9.749, *p* = 0.005; lateral: *F*(1,20) = 5.228, *p* = 0.033]. This effect was qualified by interactions with electrode location. At the midline, there was an interaction between Prime and Electrode [midline: *F*(2,40) = 10.960, *p* = 0.001], for which follow-up analyses revealed that primed sentences were less negative-going than unprimed sentences at Cz [*F*(1,20) = 9.268, *p* = 0.006] and Pz [*F*(1,20) = 19.620, *p* < 0.001]. At lateral sites, there was a significant four-way interaction between Prime, Half, Hemisphere, and Anteriority [*F*(1,20) = 9.015, *p* = 0.007], but the three-way interactions were not significant (all *p*s > 0.25). However, there was a significant two-way interaction between Prime and Anteriority [lateral: *F*(1,20) = 17.484, *p* < 0.001], for which follow-up analyses revealed that primed sentences were less negative than unprimed sentences at posterior sites [lateral: *F*(1,20) = 14.760, *p* = 0.001]. There was a main effect of Half such that the ERPs were more negative during the second half than the first half [midline: *F*(1,20) = 7.077, *p* = 0.015; lateral: *F*(1,20) = 3.662, *p* = 0.070], but this did not interact with the priming manipulation. Thus, at the main verb, there was a widespread N400 effect for unprimed as compared to primed sentences that extended into the late 500–900 ms time window. This prime-related modulation of the N400 did not change over the course of the experiment.

### Correlation Between Production-to-Comprehension and Comprehension-to-Production

Correlations were run between the significant Syntactic Choice priming effect and the ERP priming effect (at Fz, Cz, Pz electrodes and LF, RF, LP, and RP ROIs, for the 300–500 ms, and 500–900 ms windows). No significant correlations were found (all *p*s > 0.05).

## Discussion

This study presents a novel cross-modality structural priming paradigm from production to comprehension, and compared it to cross-modal comprehension-to-production priming in a within-participant design, to examine the relationship between production and comprehension. Recent models propose that production and comprehension share underlying processing mechanisms (Pickering and Garrod, 2004, 2013; MacDonald, 2013; Dell and Chang, 2014). Previous research on cross-modality priming from comprehension to production (e.g., Bock et al., 2007) has provided evidence for a link between the modalities in the one direction. However, less well-known is whether the same relationship holds for priming in the other direction, from production to comprehension. This study is the first to examine this question using a temporally sensitive measure (ERP) of comprehension processing and it also includes the more standard comprehension-to-production direction of priming for comparison. The *Comprehension-to-Production* priming task revealed the expected effect of priming in terms of syntactic choice, such that more passive target descriptions were made following passive, as compared to active, prime sentences. The novel *Production-to-Comprehension* priming task revealed a reduced N400 effect in response to primed, as compared to unprimed, passive sentences, suggesting that the mechanisms underlying production and comprehension are linked while engaged in processing both modalities, lending further support for theoretical models that describe mechanisms that link production and comprehension processes. Though no explicit correlations were found between the two tasks, the differences in granularity of the response measures (binary Syntactic Choice and fine-grained ERPs) may preclude the observation of such a relationship. Nevertheless, the findings of priming across the modalities at all, and in both directions of priming, provides strong evidence of a link between the modalities.

First, turning to the more commonly studied cross-modality structural priming direction, *Comprehension-to-Production* priming, a priming effect was found in terms of syntactic choice. No effect of priming emerged in the speed of response measures – RT or average syllable duration – for passive or active target responses. The syntactic choice effect replicates previous studies of within-modality production priming (e.g., Bock, 1986; Mahowald et al., 2016) and cross-modality priming into production (Bock et al., 2007). In contrast, the few previous studies that have examined RT in production priming have found priming effects (Corley and Scheepers, 2002), albeit of a small magnitude (Pickering and Ferreira, 2008) or not aligning with the pattern observed in syntactic choice (Segaert et al., 2011). More specifically, Segaert et al. (2011) found a syntactic choice priming effect for passive responses, but no corresponding priming effect in RTs. Only after the relative frequency of passives was boosted in a training block did they observe RT priming for passives. They concluded that unlike syntactic choice priming, which shows an inverse frequency effect (Chang et al., 2006; Jaeger and Snider, 2008), RT priming is found for the more frequent structure. In the current study, then, it is not surprising that no RT priming was found for the less-frequent passive structures. However, we also did not find an effect of priming on RTs for the more frequent active structure. In addition to RTs, the current study also measured participants’ average syllable duration, and found no effect of priming for either passive or active target structures, in parallel to the RT data. Importantly, the RT measure did show an effect of task order: As expected, passive and active sentences were produced more quickly as the experiment unfolded, suggesting that the null priming effect observed in the RT measure is not simply an effect of measurement (in)sensitivity. Thus, given the inconsistency in latency priming effects in the studies conducted so far, and the relatively small number of studies collecting latency measures alongside syntactic choice, further research is needed to understand the contexts that lead to these variable priming effects in production.

The other cross-modal priming direction, priming from production to comprehension, has been addressed in only two published studies (to our knowledge), one of which used an offline sentence-picture matching task (Branigan et al., 2005) and one from the perspective of neural substrates rather than online processing (Segaert et al., 2012). Previous studies of comprehension-to-comprehension priming using ERPs have typically found reductions in the P600 (Ledoux et al., 2007; Tooley et al., 2009, 2013; Boudewyn et al., 2014; for a review, see Tooley and Traxler, 2010), though all studies examined comprehension-to-comprehension priming using visual presentation. The current study, the first to study cross-modality structural priming into comprehension with ERPs and the first ERP comprehension priming study to use auditory presentation, found a priming effect in terms of a reduction of the N400, and no P600 effect. Note that this negativity extends into the later time window and is more temporally diffuse than traditional N400 effects. This pattern is common for ERP effects evoked by auditorily -rather than visually presented stimuli (e.g., Holcomb and Neville, 1991; Kutas and Federmeier, 2011; Grey and Van Hell, 2017). The traditional view holds that the P600 indexes syntactic processing (e.g., Osterhout and Holcomb, 1992; Hahne and Friederici, 1999; Tanner et al., 2017) and the N400 indexes lexico-semantic access and integration (e.g., Kutas and Hillyard, 1980). In recent years, this categorical distinction has been challenged in light of studies that reported P600 effects in response to semantic manipulations (for a review, see Kuperberg, 2007), and that P600s show sensitivity to manipulations typical of a P300 experiment (i.e., sensitive to the probability and saliency of the syntactic structure, Coulson et al., 1998; see also, Van Petten and Luka, 2012), and that N400s have been found in response to grammatical manipulations. In two structural priming studies, N400 effects were found on the repeated verbs in primed sentences and P600s effects were found to words disambiguating the structure (Ledoux et al., 2007; Tooley et al., 2009). That is, priming was investigated in reduced relative clause and main clause sentences, where the verb appeared prior to a disambiguating phrase (e.g., “*The speaker proposed by the group …*” vs. “*The speaker proposed the solution …*”). These effects were interpreted as separately reflecting lexical repetition (N400) and syntactic priming (P600). In the current study with active and passive sentences, it was the main verb (e.g., “*helping*” vs. “*helped*”) that cued the syntactic structure of the sentence. Like previous studies, lexical overlap was included in the verb across prime and target sentences, thus our N400 effects at the verb may reflect both lexical and syntactic priming effects, and, therefore, we choose to refer to the present effect as structural priming (see also, Pickering and Ferreira, 2008).

Why else might the N400, rather than the P600, be expected in response to structural priming in this case? First, the verb was repeated in prime/target pairs to enhance priming (this lexical overlap is often needed for comprehension priming to emerge, see Tooley and Traxler, 2010). Thus, lexical priming, which typically elicits an N400 effect (Besson and Kutas, 1993), may influence the syntactic priming in this task. Indeed, earlier positivities, between 300 and 500 ms (Ledoux et al., 2007) as well as reduced N400s (in the context of repeated verbs, Ledoux et al., 2007; Tooley et al., 2009) have also been found in priming experiments. Future studies should examine production-to-comprehension priming using prime/target pairs with and without verb overlap to tease apart aspects of lexical priming from syntactic priming. Second, supporting the idea that the N400 may reflect other aspects of language processing, beyond the classical idea of strictly indexing lexico-semantic access and integration, N400s have been found to index grammatical processing in various populations, suggesting that, at least at times, it may reflect grammatical processing: in second language processing, particularly in the early stages of processing (Morgan-Short et al., 2012), but also in proficient bilinguals (Tanner et al., 2014); in monolinguals when individual variation is examined (Tanner and Van Hell, 2014); and in left-handers (Grey et al., 2017). Third, most ERP studies of syntactic priming used more complicated syntactic structures (e.g., main clause versus relative clause sentences; e.g., Ledoux et al., 2007) than used here. Though passive sentences are more syntactically complex than active sentences (Mack et al., 2013), they may not require the same degree of complex syntactic processing as relative clause sentences. Fourth, it is possible that the N400 reflects semantic integration difficulty related to thematic role reanalysis. That is, when participants hear “*The girl was*,” they are expecting an active sentence. When the main verb “*helped*” is presented, not only is syntactic reanalysis to a passive structure required, but it is also necessary to reassign “*the girl*” from an agent role to a theme role. Difficulty related to this thematic role reanalysis and integration may be reduced for primed, as compared to unprimed sentences. Fifth, as discussed in the Introduction, Dell and Chang’s (2014) P-Chain model does not predict structural priming from production-to-comprehension, because error-based learning, their proposed mechanism of structural priming, only occurs during comprehension. In recent work, Fitz and Chang (in press) expanded on this idea in their model of ERPs as a function of error-based learning where they claim that structural priming that affects the underlying syntactic processing should modulate the syntactic P600 effect. As stated, for the current study, the P-Chain model predicts that there should be no learning on the production prime trial. This would not lead to any associated changes in the comprehension system on the target trial, and no modulation of the syntactic P600 should be found. An alternative interpretation for the observed N400 effect comes from Coulson et al. (1998), who found that N400s, in addition to P600s, were sensitive to probability manipulations of ungrammatical stimuli within an experiment. This effect was modeled by Fitz and Chang (in press) as a function of learning, where ungrammatical items lead to more learning than grammatical items because ungrammatical items generate more error due to being less expected. In the current study, the passive prime sentences are grammatical, but since they are relatively infrequent in general language use, are less expected items than active sentences. Therefore, more learning should follow the passive prime sentences than active prime sentences. This learning is observed on the N400 component, rather than the P600 component, because the passive sentences do not contain grammatical errors so do not generate a syntactic P600 effect. Finally, priming is comprised of both explicit and implicit processing (Chang et al., 2012). Participants’ explicit linking of similar lexical forms across prime/target pairs may contribute to the production-to-comprehension priming effect.

Even though multiple accounts are possible for why the pre-sent study observed an N400 in production-to-comprehension priming, the significant N400 priming effect does signify an interaction between the modalities in the direction of production-to-comprehension in this online task. As mentioned, this finding is not predicted by the P-Chain model (Dell and Chang, 2014). One way the P-Chain model may account for this effect is by postulating that the priming stems from error-based learning of participants’ comprehension of their own productions that leads to the effect on target comprehension processing. This might occur despite the fact that passives are not ungrammatical, and may relate to the inverse frequency account as discussed just above. However, the finding of priming in both directions between production and comprehension is consistent with Pickering and Garrod’s Interactive Alignment model (2004) and their integrated account of production and comprehension Pickering and Garrod (2013), if the forward prediction model processing can impact the underlying processing, as well as findings that production and comprehension rely on similar processing mechanisms (Kempen et al., 2012).

Another final point to note is that many studies of structural priming have focused on the effect of distance, or lag, between prime and target sentences, particularly in the debate between models of structural priming (i.e., the implicit learning account, Chang et al., 2006 and the residual activation account, Pickering and Branigan, 1998). Though the materials of the current study included prime-target pairs separated by 0, 1, or 2 filler sentences, the task was not explicitly designed to test this lag effect. Specifically, in the production-to-comprehension direction, the number of trials at each distance is not sufficient for studying this specific lag question, and the range of distances included is not sufficiently variable to detect large effects (e.g., to properly assess distance, lags of up to 10 would be preferable). Moreover, this lag account is often tested along with the lexical boost account (e.g., Hartsuiker et al., 2008), examining how the repetition of verbs across prime/target pairs influences priming, which requires conditions with and without repetition, of which the current study only included trials with repeated verbs. Given this study’s finding of an influence of production on comprehension, though, a relevant question for future studies would be to explicitly examine whether and how distance between prime/target pairs and lexical repetition influence production-to-comprehension priming.

To conclude, this study presents a novel paradigm for investigating the relationship between production and comprehension. Moreover, the present production-to-comprehension cross-modality structural priming task is the first to examine priming in this direction using a temporally sensitive online neural measure of comprehension processing, and is presented in conjunction with a comprehension-to-production priming task to investigate whether the link between the two modalities is evident in both directions. Significant cross-modality priming was found in both priming directions, extending and strengthening previous structural priming findings and supporting theories proposing linked processing and representations between production and comprehension (Pickering and Garrod, 2004, 2013; MacDonald, 2013; Dell and Chang, 2014). Though no explicit correlation was found between the priming effects from each task in this study, future studies should use tasks with more similar outcome measures so that correlational measures may serve as an index of the relationship between the modalities. The novel use of EEG for cross-modality priming into comprehension, using auditory ERPs, provides an online measure of processing and can be used for further investigation of the relationship between production and comprehension at different linguistic levels (e.g., lexico-semantic or phonological processing). The use of both directions of cross-modality priming allows for the exploration of the influence of extra-linguistic factors during natural conversation, such as accented speech, or the development of the production and comprehension relationship in first and second language development. Given that individuals use both production and comprehension in natural language use (Hasson et al., 2018), future research and more models of language processing should take into account both production and comprehension and how the two interact in different linguistic and sociocultural discourse contexts.

## Ethics Statement

This study was carried out in accordance with the recommendations of the Pennsylvania State University’s Office for Research Protection with written informed consent from all subjects. All subjects gave written informed consent in accordance with the Declaration of Helsinki. The protocol was approved by the Pennsylvania State University Institutional Review Board.

## Author Contributions

KL and JvH contributed to the conception and design of the study. KL organized the data and performed the statistical analysis. KL wrote the first draft of the manuscript. Both authors contributed to the manuscript revision, read, and approved the submitted version.

## Conflict of Interest Statement

The authors declare that the research was conducted in the absence of any commercial or financial relationships that could be construed as a potential conflict of interest.

## Footnotes

1 This task was run as a control for a parallel study on dialogue with a foreign-accented speaker (Litcofsky, 2017) and will not be discussed further here.

2 Analyses were also run where Order was operationalized as simply the trial number, since the number of actives or passives comprehended is not dissociable from trial number. The pattern of results are qualitatively the same across analyses.

## APPENDIX

**Table 7:**

**Critical stimuli – transitives**			
Actors			
Boy	King	Priest	Thief
Bride	Knight	Queen	Waiter
Chef	Man	Sailor	Witch
Fireman	Nun	Soldier	Wizard
Girl	Nurse	Swimmer	Woman
Verbs			
Burn	Embrace	Massage	Spray
Bury	Enchant	Measure	Startle
Call	Follow	Paint	Stop
Carry	Grab	Pay	Strangle
Chase	Greet	Photograph	Tackle
Comfort	Help	Pull	Tease
Cover	Interview	Punch	Threaten
Crush	Kick	Push	Tickle
Drag	Kiss	Scare	Warn
Dress	Lift	Serve	Watch
**Filler stimuli – locatives**			
Objects			
Anchor	Chair	Jacket	Saxophone
Apple	Celery	Key	Scarf
Arrow	Circle	Kite	Shell
Ax	Corn	Knife	Shirt
Bag	Crown	Lamp	Shoe
Balloon	Cup	Leaf	Shovel
Banana	Doorknob	Lock	Sock
Barrel	Dress	Magnet	Spoon
Basket	Drum	Mirror	Square
Bell	Envelope	Mop	Stamp
Belt	Fence	Pan	Star
Bomb	Flag	Paperclip	Syringe
Book	Flashlight	Peanut	Tape
Bottle	Flower	Pear	Teapot
Bow	Football	Pencil	Telephone
Bowl	Fork	Piano	Tie
Box	Glass	Pineapple	Tomato
Brush	Globe	Pitcher	Trumpet
Bucket	Guitar	Present	Umbrella
Button	Gun	Razor	Vest
Cake	Hammer	Ring	Wallet
Camera	Hanger	Robot	Wheel
Can	Harp	Ruler	Wrench
Candle	Hat	Sandwich	Yoyo
Carrot	Iron	Saw	Zipper
Prepositions			
Above	Beneath	Next to	Under
Against	Beside	On	With
Behind	In front of	On top of	
Below	Near	Over	
Deer	Gorilla	Pig	
Dinosaur	Horse	Rabbit	
Dog	Kangaroo	Raccoon	
**Filler stimuli – intransitives**			
Animals			
Alligator	Dolphin	Leopard	Rhinoceros
Bear	Donkey	Lion	Seahorse
Bee	Dragon	Lobster	Skunk
Bird	Duck	Monkey	Snail
Butterfly	Eagle	Moose	Squirrel
Camel	Elephant	Mouse	Tiger
Cat	Fish	Owl	Turkey
Chicken	Fox	Panda	Turtle
Cow	Frog	Parrot	Wolf
Crab	Giraffe	Penguin	Zebra
Verbs			
Bite	Drink	Fight	Play
Die	Eat	Move	Sleep
