# Supplementary material for: Bi-Directional Evidence Linking Sentence Production and Comprehension: A Cross-Modality Structural Priming Study
Source: Front Psychol. 2019 May 28;10:1095. doi: 10.3389/fpsyg.2019.01095 (PMC6546884; doi:10.3389/fpsyg.2019.01095)
Supplement: Supplementary file 1 [file Data_Sheet_1.docx]

**Linking production and comprehension: A cross-modality structural priming study of sentence processing**

***Supplemental Material***

*Production-to- Comprehension Results*

Auxiliary verb before the main verb: ‘was’. Grand mean waveforms for the word ‘was’, the word before the main verb, for primed (passive prime – passive target) and unprimed (active prime – passive target) conditions are plotted in Figure 10. Visually, there is a reduced late negativity to primed sentences as compared to unprimed sentences. In the 300 – 500 ms time window, there were no differences between the conditions (all *p*s > .19). In the 500 – 900 ms time window, there was a main effect of Prime such that primed sentences were less negative than unprimed sentences (midline: *F*(1, 20) = 6.541, *p* = .019; lateral: *F*(1, 20) = 3.762, *p* = .067). This effect was qualified by interactions with topography. At midline sites, there was an interaction of Prime and Electrode (midline: *F*(2, 40) = 7.232, *p* = .009), for which follow-up analyses revealed that primed sentences were less negative than unprimed sentences at Cz (*F*(1, 20) = 8.113, *p* = .01) and Pz (*F*(1, 20) = 14.310, *p* = .001). At lateral sites, there was an interaction of Prime and Anteriority (*F*(1, 20) = 6.285, *p* = .021), for which follow-up analyses revealed that primed sentences were less negative than unprimed sentences at posterior sites (*F*(1, 20) = 10.360, *p* = .004). There were no effects of Half (all *p*s > .16)). Thus, at the word ‘was’ there was a late negativity to unprimed, as compared to primed sentences, and this effect was most pronounced at central and posterior sites.


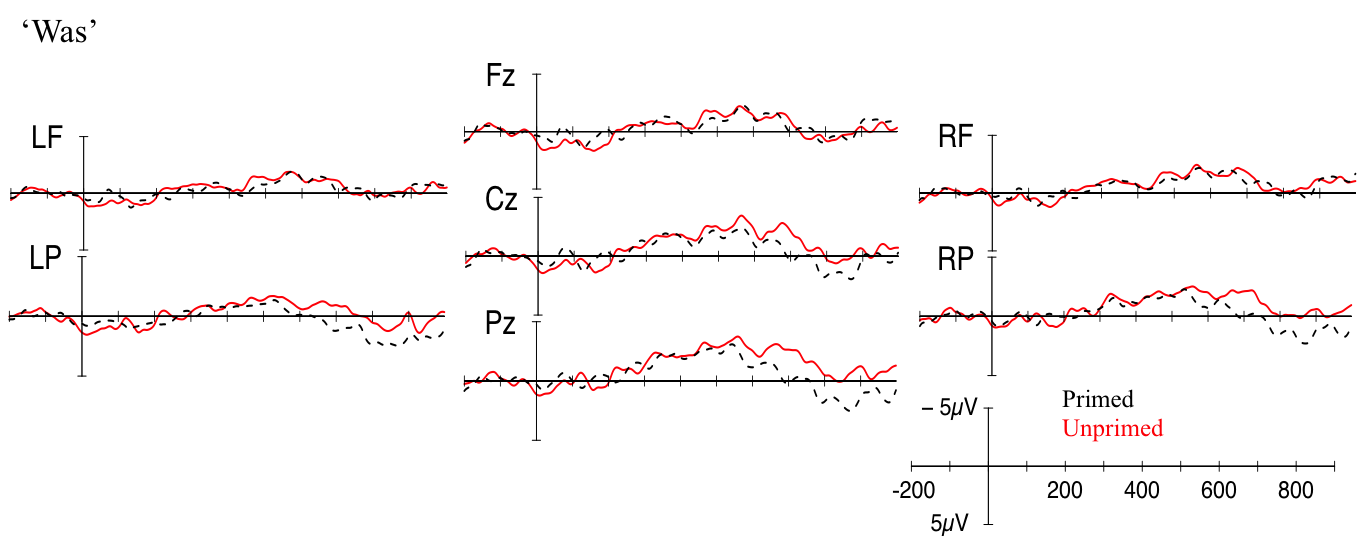


Figure 10. Grand mean waveforms for the word ‘was’ for primed (passive prime – passive target; black dotted line) and unprimed (active prime – passive target; red solid line) conditions. Onset of the main verb is indicated by the vertical bar. The calibration plot shows amplitude is plotted on the y-axis (negative plotted up). Time is plotted on the x-axis; each tick mark indicates 100 ms. LF=left frontal; RF=right frontal; LP=left posterior; RP=right posterior.

Word after the main verb: ‘by’. Grand mean waveforms for the word ‘by’, the word after the main verb, for primed (passive prime – passive target) and unprimed (active prime – passive target) conditions are plotted in Figure 11. Visually, there are no differences between the conditions. In the 300 – 500 ms time window, there were two marginal interactions at lateral sites (Prime x Half x Hemisphere x Anteriority: *F*(1, 20) = 3.723, *p* = .068; Prime x Anteriority: *F*(1, 20) = 4.018, *p* = .059), for which follow-up analyses revealed no significant effects (all *p*s > .40). There were no other differences between the conditions (all *p*s > .25).


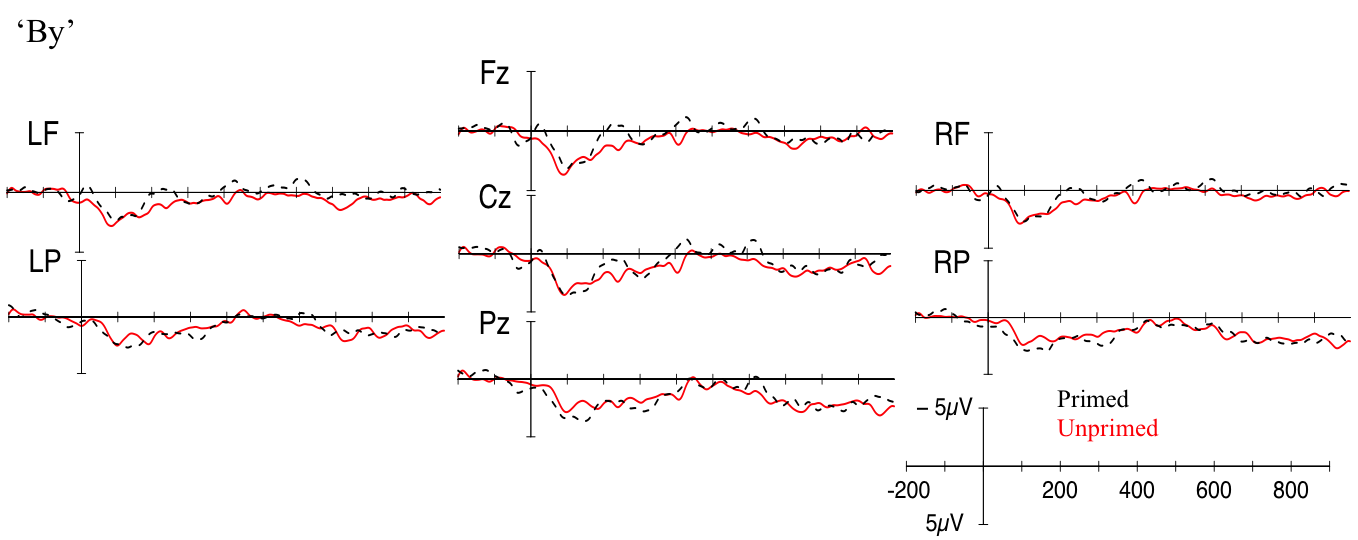
Figure Figure 11. Grand mean waveforms for the word ‘by’ for primed (passive prime – passive target; black dotted line) and unprimed (active prime – passive target; red solid line) conditions. Onset of the main verb is indicated by the vertical bar. The calibration plot shows amplitude is plotted on the y-axis (negative plotted up). Time is plotted on the x-axis; each tick mark indicates 100 ms. LF=left frontal; RF=right frontal; LP=left posterior; RP=right posterior.

In the 500 – 900 ms time window, there was an effect of Half such that ERPs in the second half of the experiment were more negative than in the first half of the experiment (midline: *F*(1, 20) = 4.925, *p* = .038; see Figures 12 and 13 for grand mean plots of the word ‘by’ in the first and second halves, respectively). There was also an interaction between Half and Anteriority (lateral: *F*(1, 20) = 4.100, *p* = .056), but follow-up analyses were not significant (all *p*s > .18). There were no effects of Prime (all *p*s > .12). Thus, ERPs time-locked to the word ‘by’ revealed no differences between primed and unprimed sentences, but, overall, ERPs were more negative in the second than in the first half of the experiment.


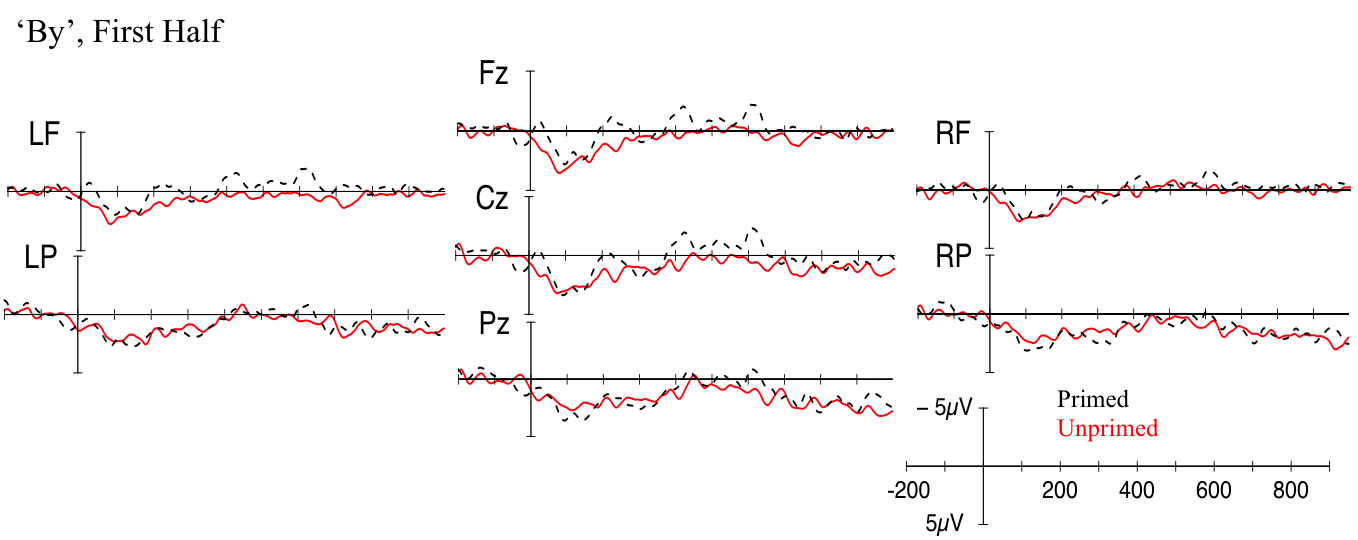


Figure 12. Grand mean waveforms from the first half of the experiment for the word ‘by’ for primed (passive prime – passive target; black dotted line) and unprimed (active prime – passive target; red solid line) conditions. Onset of the main verb is indicated by the vertical bar. The calibration plot shows amplitude is plotted on the y-axis (negative plotted up). Time is plotted on the x-axis; each tick mark indicates 100 ms. LF=left frontal; RF=right frontal; LP=left posterior; RP=right posterior.


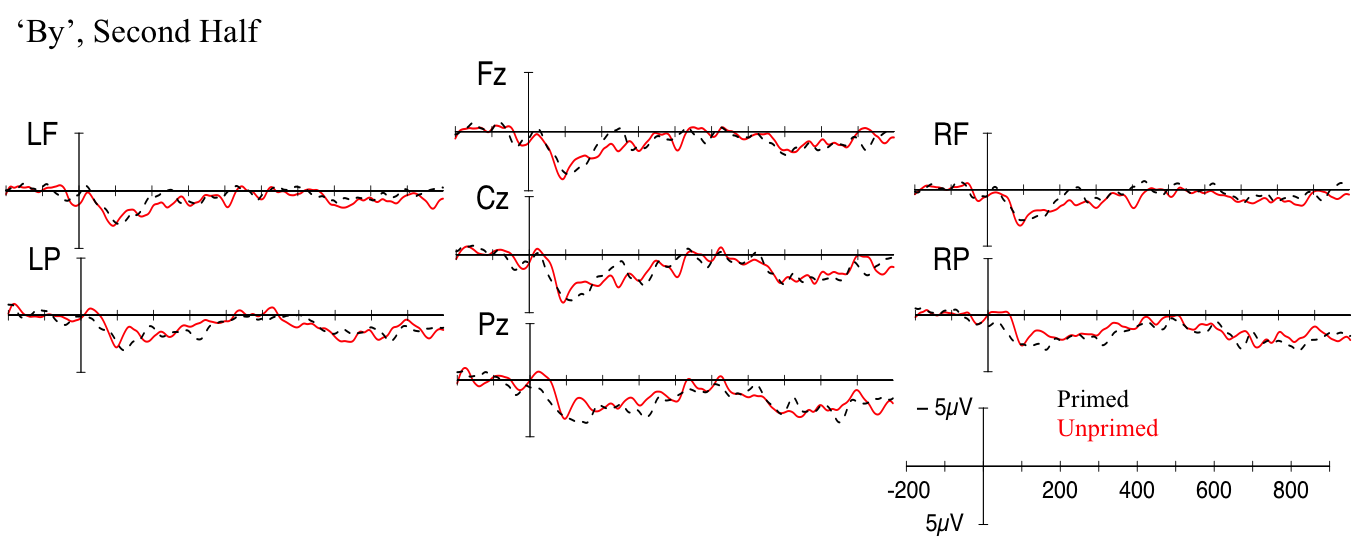


Figure 13. Grand mean waveforms from the second half of the experiment for the word ‘by’ for primed (passive prime – passive target; black dotted line) and unprimed (active prime – passive target; red solid line) conditions. Onset of the main verb is indicated by the vertical bar. The calibration plot shows amplitude is plotted on the y-axis (negative plotted up). Time is plotted on the x-axis; each tick mark indicates 100 ms. LF=left frontal; RF=right frontal; LP=left posterior; RP=right posterior.

Three words after the main verb: second noun. Grand mean waveforms for the second noun, three words after the main verb, for primed (passive prime – passive target) and unprimed (active prime – passive target) conditions are plotted in Figure 14. Visually, there are no differences between the conditions. In the 300 – 500 ms time window, there was a marginal interaction between Prime and Electrode (midline: *F*(2, 40) = 3.320, *p* = .077), but follow-up analyses were not significant (all *p*s > .12). None of the main or interaction effects reached significance (all *p*s > .24). In the 500 – 900 ms time window, there was an interaction of Prime and Electrode (midline: *F*(2, 40) = 7.406, *p* = .007), and follow-up analyses revealed that primed sentences were less negative than unprimed sentences at Fz (*F*(1, 20) = 4.55, *p* = .045). There were no effects of Half (all *p*s > .58). Thus, the second noun in the sentence showed a late frontal negativity to unprimed as compared to primed sentences.


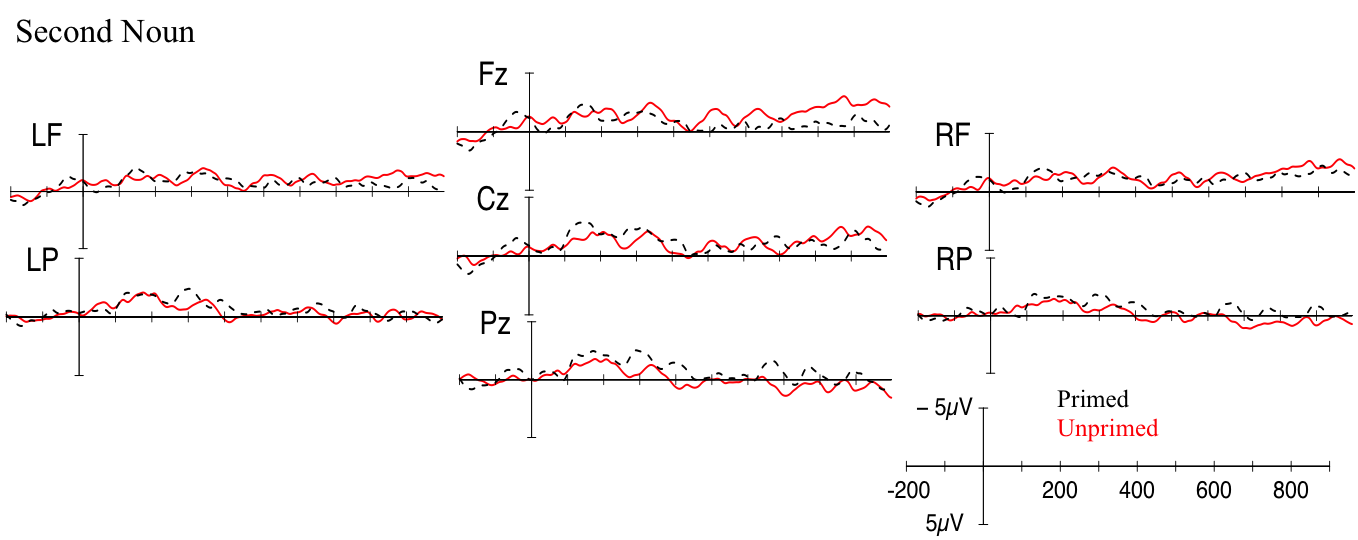


Figure 14. Grand mean waveforms for the second noun phrase for primed (passive prime – passive target; black dotted line) and unprimed (active prime – passive target; red solid line) conditions. Onset of the main verb is indicated by the vertical bar. The calibration plot shows amplitude is plotted on the y-axis (negative plotted up). Time is plotted on the x-axis; each tick mark indicates 100 ms. LF=left frontal; RF=right frontal; LP=left posterior; RP=right posterior.
